# Constitutive nuclear factor-kappa B mRNA, protein overexpression and enhanced DNA-binding activity in thymidylate synthase inhibitor-resistant tumour cells

**DOI:** 10.1038/sj.bjc.6600753

**Published:** 2003-02-18

**Authors:** W Wang, J Cassidy

**Affiliations:** 1 Department of Medicine and Therapeutics, Institute of Medical Sciences, University of Aberdeen, Foresterhill, Aberdeen AB25 2ZD, UK; 2 Department of Medical Oncology, University of Glasgow, Switchback Road, Glasgow G61 1BD, UK

**Keywords:** NF-*κ*B, I*κ*Bα, 5-FU, tomudex, chemoresistance

## Abstract

In this study, the gene copy number, mRNA and protein expression levels and nuclear DNA-binding activity of nuclear factor kappa B (NF-*κ*B) were compared in a panel of five pairs of thymidylate synthase (TS) inhibitor-resistant and wild-type parent cancer cell lines. High constitutive NF-*κ*B DNA-binding activity was detected in all chemoresistant cell lines. The upregulated NF-*κ*B activity was composed of NF-*κ*B subunits p50 and p65. Four out of five resistant cell lines constitutively overexpressed NF-*κ*B p50 and p65 mRNA and protein. One resistant cell line with the highest NF-*κ*B DNA-binding activity showed normal p50 and p65 protein expression. No NF-*κ*B gene amplification was detected in resistant cell lines. Transient exposure of wild-type RKO_WT_ and H630_WT_ cells to 5-FU induced NF-*κ*B DNA-binding activity but had no effect on NF-*κ*B protein expression in these cells. Our results indicate that high constitutive NF-*κ*B activity caused by its gene overexpression is an intrinsic character of TS inhibitor-resistant cells. NF-*κ*B can antagonise anticancer drug-induced apoptosis. High NF-*κ*B expression and nuclear activity in TS inhibitor-resistant cancer cells may play an important role in the chemoresistance.

The production of the pyrimidine nucleotide TMP from deoxyuridine monophosphate is a critical step in the *de novo* pathway of DNA synthesis. Since this reaction is catalysed by thymidylate synthase (TS, EC 2.1.1.45) using folate cosubstrate 5, 10-methylene tetrahydrofolate and is the only source of *de novo* cellular thymidylate, it makes TS an attractive molecular target for cytotoxic drugs (Jackman and Calvert, 1995; Rustum *et al*, 1997). TS inhibitors (5-fluorourocil (5-FU) and tomudex (TDX)) block TS activity and have been effectively used in the treatment of human solid tumours such as colorectal, breast and lung cancers (Burris *et al*, 1994; Cunningham *et al*, 1994; Smith *et al*, 2002). As with other anticancer drugs, acquired or inherent resistance to these drugs is still one of the main barriers for their clinical use. Apart from high levels of TS protein expression (Jackman *et al*, 1995), the other molecular mechanisms of TS-inhibitor-resistance are still largely unknown.

Nuclear factor-kappaB (NF-*κ*B) is a transcription factor that is composed of five subunits (p50/p105, p52/p100, p65 (RelA), RelB and c-Rel). NF-*κ*B can bind to DNA target sites (*κ*B sites) and influence downstream gene expression (Perkins, 2000). The inhibitor of *κ*B (I*κ*B) is a natural inhibitor of NF-*κ*B. In most normal cells, I*κ*B binds to NF-*κ*B and retains NF-*κ*B in the cytoplasm as an inactive complex. Many chemical and biological stimuli can induce I*κ*B phosphorylation, ubiquitinisation and subsequent degradation. NF-*κ*B will be released from the NF-*κ*B–I*κ*B complex and translocated into the nucleus. The liberated NF-*κ*B then binds to the promoter region of the relevant downstream genes and triggers a series of transcriptional events (Karin, 1999).

Some anticancer drugs, cytokines and radiation can induce NF-κB activity in cancer cells (Baldwin Jr, 1996; Pahl, 1999). NF-κB antagonises the cytotoxicity of some anticancer drugs by inducing the expression of anti-apoptotic genes (e.g. *c-IAPs, IXAP, A1/Bfl-1* and *IEX-IL*) (Wang *et al*, 1998,1999b; Wu *et al*, 1998). Human cancer cells with induced NF-*κ*B nuclear activity have demonstrated resistance to apoptosis induced by chemotherapy or radiotherapy (Barkett and Gilmore, 1999; Rayet and Gelinas, 1999; Baldwin Jr, 2001; Yamamoto and Gaynor, 2001). Transfection of mutant super-repressor IκBα into tumour cell lines can inhibit NF-κB activity and enhance the cytotoxicity of anticancer drugs *in vitro* (Wang *et al*, 1996) and *in vivo* (Wang *et al*, 1999a; Cusack *et al*, 2001).

In this study, nuclear NF-*κ*B activity, the protein and mRNA expression levels of NF-*κ*B in five pairs of TS inhibitor-resistant and sensitive cell lines were tested. The results demonstrate that constitutive high nuclear NF-*κ*B activity and overexpression of NF-*κ*B protein and mRNA are intrinsic characteristics of TS inhibitor-resistant cell lines.

## MATERIALS AND METHODS

### Cell culture

Three TDX-resistant cell lines (colorectal tumour: H630_TDX_ and RKO_TDX_; lymphoblastoid: W1L2_TDX_), two 5-FU-resistant colorectal cancer cell lines (H630_5-FU_ and R10_5-FU_) and the relevant parent cell lines were chosen for this study (The chemoresistant cell lines were kindly provided by Professor PG Johnston, Department of Oncology, The Queen's University of Belfast). The characteristics and culturing conditions have been described previously (Boonsong *et al*, 2000; Wang *et al*, 2001). To rule out induction of NF-*κ*B by the maintenance dose of 5-FU/TDX, all drug-resistant cells were cultured in drug-free RPMI 1640 medium supplemented with 10% fetal calf serum, 50 U ml^−1^ penicillin, 50 *μ*g ml^−1^ streptomycin for 10 days before harvesting by trypsinisation for further analysis.

### Electrophoretic mobility-shift assays (EMSA)

The nuclear and cytoplasmic protein extraction and EMSA were carried out as previously described (Wang *et al*, 1996). The nuclear extract (5 *μ*g) was incubated with 1 *μ*g poly(dIdC) (Sigma-Aldrich, Dorset, UK) in binding buffer (50 mM Tris (pH 7.6), 250 mM KCl, 25 mM DTT, 5 mM EDTA and 25% glycerol) for 10 min at room temperature (RT). Approximately 20 000 c.p.m. of ^32^P-labelled 22-mer double-stranded NF-κB DNA probe (5′-AGTTGAGGGGACTTTCCCAGGC-3′) was added and incubated at RT for 20 min. Oct-1 (5′-TGTCGAATGCAAATCACTAGAA-3′) was used as loading control for EMSA. The EMSA conditions of Oct-1 were the same as those for NF-*κ*B. For supershift assay and binding specificity determination, 5 *μ*g of nuclear extract mixture from different resistant cell lines was incubated with 0.4 *μ*g 2 *μ*l^−1^ of NF-*κ*B p65 or p50 antibody (Santa Cruz Biotechnology, Santa Cruz, CA, USA) or 20×wild-type or mutant (5′-AGTTGATATTACTTTTATAGGC-3′) unlabelled NF-*κ*B probe for 30 min before EMSA analysis. The complexes were separated on a 6% polyacrylamide gel and exposed for autoradiography.

### Western blot analysis

The cytoplasmic protein (40 *μ*g lane^−1^) was electrophoresed through a 10% SDS–PAGE and transferred to a PVDF membrane (Millipore, UK). The blots were stained with rabbit polyclonal primary (p50, 1 : 500; p65 and I*κ*B*α*, 1 : 200; Santa Cruz Biotechnology, Santa Cruz, CA, USA) and HRP-conjugated donkey anti-rabbit secondary (1 : 5 000; Amersham Pharmacia Biotech, NJ, USA) antibodies, respectively, for 1 h at RT. The loading quantity of protein was verified by staining the same membranes with monoclonal anti-*α*-tubulin or antivinculin antibody (Sigma-Aldrich, Dorset, UK; anti-*α*-tubulin: 1 : 2000; antivinculin: 1 : 1000) and HRP-conjugated sheep anti-mouse secondary antibody (1 : 5000; Amersham Pharmacia Biotech, NJ, USA). The signal was detected using an ECL Western blotting detection kit (Amersham Pharmacia Biotech, NJ, USA). The intensity of the bands was scanned and analysed using Molecular Analyst software (Bio-Rad Laboratories, CA, USA). This programme was also used to analyse the band intensity of Northern and Southern blots.

### Northern blot analysis

Total RNA was extracted using the previously described method (Wang *et al*, 2001). Briefly, cells (∼5×10^6^) were digested in denaturing buffer and protein was extracted twice with phenol (pH 4.3) : chloroform (1 : 0.3). Total RNA in the aqueous phase was precipitated with an equal volume of isopropanol and then washed with 70% ethanol. Total RNA (20 *μ*g) from drug-sensitive and resistant cells was electrophoresed through a 1% agarose gel containing 1.9% formaldehyde. After transfer to a Hybond-N^+^ nylon membrane (Amersham), the blotted RNA was immobilised by exposing under 250 nm UV light for 3 min. Membranes were hybridised with [^32^P] dATP random labelled probes at 68°C overnight. The signals were detected by exposing the membranes to a FUJI Phosphor Plate overnight and scanned using a FUJI FILM-3000 Phosphor-Imager. Blots were stripped and rehybridised with different probes. NF-*κ*B p50 and p65 subunits were subjected to Northern blotting analysis and GAPDH gene was used as an internal control. The probes were reverse transcribed and amplified from 1 *μ*g RKO_TDX_ total RNA using a one-step RT–PCR kit (Promega, CA, USA). The sequences of primers are as follows: NF-*κ*B p50 (601 bp): forward 5′-CTGGTGATCGTGGAACAGCC-3′; reverse 5′-CAGAGCCTGCTGTCTTGTCC-3′; NF-κB p65 (659 bp): forward 5′-atgcgcttccgctacaagtg-3′; reverse 5′-acaatggccacttgtcggtg-3′; GAPDH (915 bp): forward 5′-AAGGTCGGAGTCAACGGATTTG-3′; reverse 5′-CTTGACAAAGTGGTCGTTGAGG-3′. The sequence of each amplified probe was confirmed by direct automated DNA sequencing.

### Southern blot analysis

The DNA was extracted using Nucleon DNA purification kit (Nucleonbiosciences, Glasgow, UK) following the supplier's instruction. A total of 20 *μ*g of DNA from each cell line was digested with *Mva*II (p50) or *Sac*I (p65) for 3 h at 37°C. The digested DNA fragments were separated through a 0.6% agarose gel. After de-naturing in 0.1 N NaOH, the DNA was blotted onto a nylon mem-brane by overnight capillary transfer and fixed under 250 nm UV light for 3 min. The probes, hybridisation and image analysis con-ditions were the same as those for Northern blotting analysis. The same membrane was stripped and hybridised to different probes.

## RESULTS

### Higher constitutive nuclear NF-*κ*B activity in TS inhibitor-resistant cells

**Figure 1:**
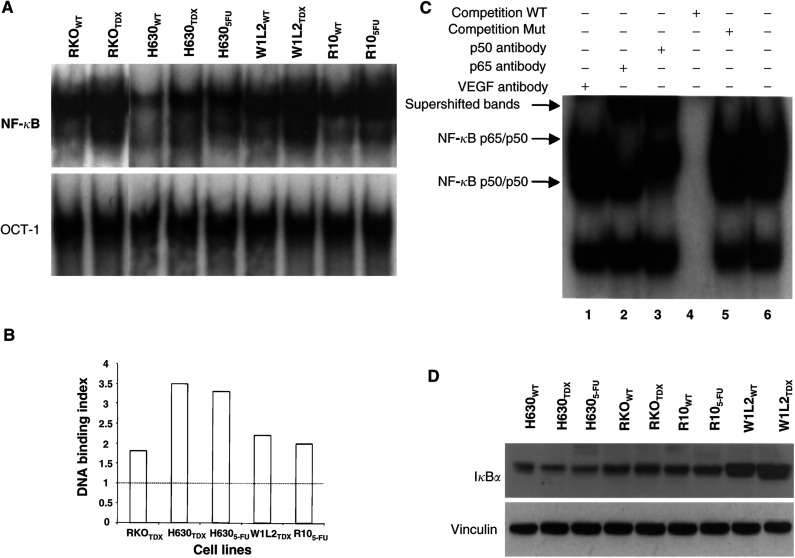
High constitutive NF-*κ*B DNA-binding activity in 5-FU- and TDX-resistant cell lines. (**A**) An equal amount (5 *μ*g) of nuclear extract was subjected to EMSA. All drug-resistant cells demonstrate higher nuclear NF-*κ*B activity. The constitutively activated transcription factor, Oct-1, was used as a protein loading control. (**B**) Ratio of NF-*κ*B DNA-binding activity between resistant and relevant parent cell lines. (**C**) Probe specificity and supershift analysis of NF-*κ*B activity in drug-resistant cells. An equal amount (5 *μ*g) of mixed nuclear extract from resistant cell lines was incubated with NF-κB p50 and p65 antibodies or unlabelled NF-*κ*B probes before EMSA analysis. (**D**) I*κ*B*α* protein levels were determined in cytoplasmic extract (40 *μ*g lane^−1^) using Western blotting analysis. The protein loading control was monitored by staining the same membrane with antivinculin antibody (lower panel).

NF-*κ*B nuclear activity was tested in five pairs of TS inhibitors (5-FU and TDX)-sensitive and -resistant cell lines. All resistant cell lines were cultured in drug-free medium for 10 days before EMSA analysis. There was substantial NF-*κ*B activity in the chemosensitive parent cell lines, but the TS inhibitor-resistant cell lines demonstrated much higher nuclear NF-*κ*B activity (Figure 1A

). The ratio of NF-*κ*B nuclear activity in paired resistant and wild-type cell lines ranged from 1.8 to 3.5 (Figure 1B). To verify the bands, 5 *μ*g of nuclear extract mixture from resistant cell lines was preincubated with 20 times higher concentration of unlabelled wild-type or mutant NF-*κ*B probe, before EMSA. The retarded bands were removed by unlabelled wild-type NF-*κ*B probe but not the mutant one (Figure 1C, lanes 4 and 5). Supershift analysis showed that NF-*κ*B p50 and p65 were involved in the constitutive NF-*κ*B activity in the resistant cells (Figure 1C, lanes 2 and 3). No supershift band was observed by using antibodies against NF-*κ*B subunits p52, c-Rel and RelB (data not shown). Anti-VEGF antibody was used as a negative control for the supershift assay. To test if the high NF-*κ*B nuclear activity in drug-resistant cells was caused by I*κ*B*α* degradation, I*κ*B*α* protein levels in drug-sensitive and -resistant cells were assessed by Western blotting analysis. It seems that I*κ*B*α* degradation was not responsible for the high constitutive NF-*κ*B nuclear activity in resistant cells because high and comparable I*κ*B*α* protein levels were detected in both resistant and relevant parent cell lines (Figure 1D).

### Constitutive overexpression of NF-*κ*B subunits p50 and p65 protein and mRNA in TS inhibitor-resistant cell lines

**Figure 2:**
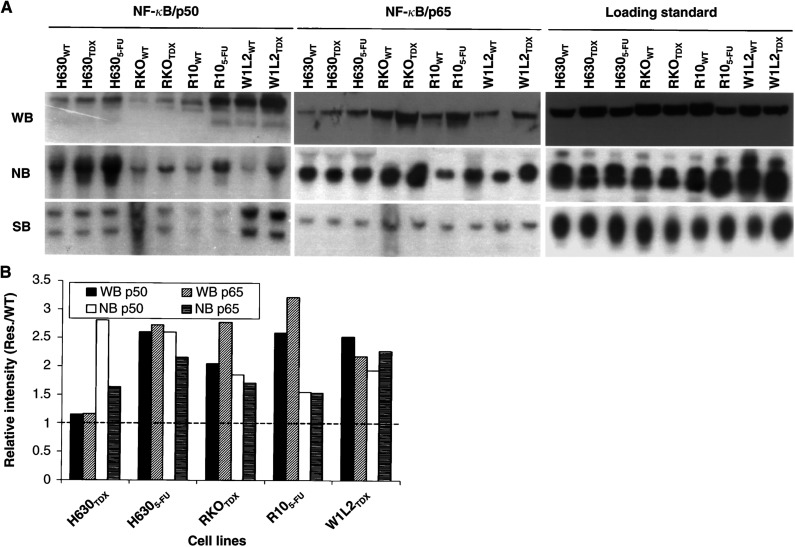
Protein and mRNA overexpression but no gene amplification of NF-*κ*B p50 and p65 in drug-resistant cell lines. (**A**) WB: Western blotting analysis of cytoplasmic extract (40 *μ*g lane^−1^) using p50 and p65 antibodies; NB: Northern blotting analysis of total RNA from different cell lines; SB: genomic DNA (20 *μ*g) was analysed by Southern blot after *Mva*II (p50) and *Sac*I (p65) cut. Loading control: tubulin for WB and GAPDH for NB and SB. (**B**) Relative protein and mRNA expression levels in resistant cell lines. The intensity of bands was analysed using Molecular Analyst software and standardised by relevant parent cells.

Constitutively upregulated transcription factor activity is frequently caused by gene overexpression (Adams and Kaelin, 1996). Therefore, we tested protein and mRNA levels of NF-*κ*B subunits p50 and p65 in drug-resistant cell lines. As shown in Figure 2

, p50 and p65 protein and mRNA were 1.5 to three-fold constitutively overexpressed in all TS inhibitor-resistant cell lines except for H630_TDX_ cells in which both proteins were only slightly upregulated. These results indicate that although high NF-*κ*B nuclear DNA-binding activity is mainly caused by enhanced nuclear translocation and DNA-binding affinity, transcriptional and translational deregulation may also play a role in the maintenance of high constitutive NF-*κ*B nuclear activity in TS inhibitor-resistant cells. We have demonstrated before that the constitutive TS gene overexpression in these cell lines was accompanied by gene amplification (Wang *et al*, 2001). We therefore tested if the high constitutive NF-*κ*B activity in these cell lines was also caused by gene amplification. Gene copy number of NF-*κ*B p50 and p65 was detected by Southern blotting analysis. As demonstrated in the bottom panel of Figure 2A, compared with relevant parent cell lines, no p50 and p65 gene amplification was detected in the drug-resistant cell lines. The change of NF-*κ*B DNA-binding activity in most drug-resistant cell lines was parallel to the increased p50 and p65 protein expression levels. In contrast, the DNA-binding activity in H630_TDX_ cells was most highly upregulated but the expression of p50 and p65 proteins in this cell line was maintained at similar levels to those of the parent cell line (Figure 2B).

### Transient exposure of cancer cells to 5-FU induced NF-*κ*B nuclear DNA-binding activity but not the expression of NF-*κ*B p50 and p65 protein

**Figure 3:**
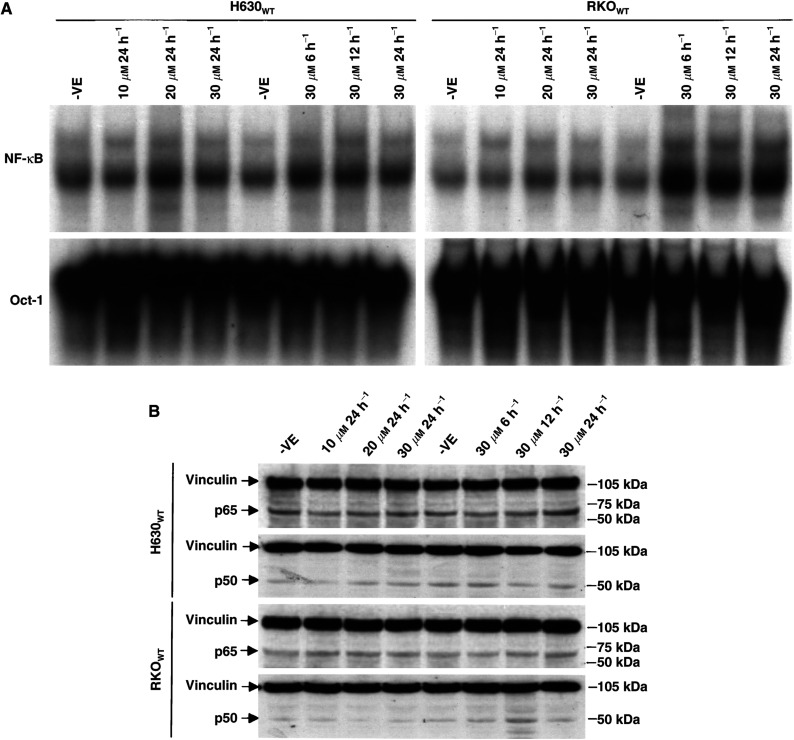
5-FU induced NF-*κ*B DNA-binding activity but had no effect on the protein expression of NF-*κ*B p50 and p65 in H630_WT_ and RKO_WT_ cells. After exposure of cancer cells to 5-FU at different concentrations and time lengths, NF-*κ*B DNA-binding activity and NF-*κ*B p50 and p65 protein expression levels were tested by EMSA (**A**) and Western blot (**B**), respectively. Oct-1 and vinculin were used as loading control for EMSA and Western blotting, respectively.

Anticancer drugs exposure can induce NF-*κ*B activity in cancer cell lines (Yamamoto and Gaynor, 2001). To test the effect of 5-FU on NF-*κ*B DNA-binding activity and protein expression levels, colorectal cancer cell lines H630_WT_ and RKO_WT_ were exposed to 5-FU at different concentrations and time lengths. Figure 3A

demonstrates that NF-*κ*B DNA-binding activity was induced by 5-FU in both a time- and concentration-dependent manner in both H630_WT_ and RKO_WT_ cell lines, whereas exposure of these cells to 30 *μ*M 5-FU up to 24 h had no effect on the expression levels of both NF-*κ*B p50 and p65 proteins in the tested cell lines.

## DISCUSSION

5-FU and TDX have been successfully used in human solid cancer chemotherapy. Both drugs inhibit TS, the enzyme converting 2′-deoxyuridine to thymidylate by reductive methylation. 5-FU can also induce metabolic disruption and cell death by incorporating into DNA and RNA. After chemotherapy, some cancer cells show acquired chemoresistance to TS inhibitors. The drug-resistant cells usually demonstrate TS protein and mRNA overexpression and TS gene amplification (Wang *et al*, 2001). Alternative molecular mechanisms of resistance to TS inhibitors have not been fully elucidated. In this study, we demonstrated high NF-*κ*B DNA-binding activity in a panel of five TS inhibitor-resistant cell lines. NF-*κ*B in resistant cell lines was composed of p65/p50 and p50/p50 dimers, which are the most common NF-*κ*B dimers and play a major antiapoptotic role in anticancer drug-treated mammalian cells (Wang *et al*, 1996,1999a; Cusack *et al*, 2000). Anticancer drugs exposure can induce NF-*κ*B activity in cancer cell lines (Yamamoto and Gaynor, 2001). The half-life of NF-*κ*B is less than 30 min and maintenance of its activity requires ongoing protein synthesis and continuous stimuli (Hohmann *et al*, 1991). To rule out the possibility that the enhanced NF-*κ*B DNA-binding activity in resistant cell lines was also induced by the maintaining dose of 5-FU or TDX, the resistant cell lines were cultured in drug-free medium for 10 days before EMSA. The cells demonstrated comparable NF-*κ*B DNA-binding activity whether or not they were cultured in the medium with or without maintenance concentrations of TS inhibitors (data not shown). In comparison with relevant parent cell lines, drug-resistant cells demonstrated higher NF-*κ*B DNA-binding activity. Thus, the high NF-*κ*B activity in drug-resistant cells is a constitutive and intrinsic feature of the resistant cell lines. NF-*κ*B is a transcription factor that antagonises apoptosis induced by some anticancer drugs (Yamamoto and Gaynor, 2001). Cancer cells with high induced NF-*κ*B activity show a high expression of antiapoptotic gene products such as *c-IAPs, IXAP, A1/Bfl-1* and *IEX-IL* (Wang *et al*, 1998). In our study, high NF-*κ*Bactivity in TS-inhibitor resistant cell lines may at least partially lead to their chemoresistance. Figure 3A demonstrates that NF-*κ*B activity could be induced by transient exposure of colorectal cancer cell lines to 5-FU. In combination with 5-FU, disulfiram, an NF-*κ*B inhibitor, strongly enhances the cytotoxicity of 5-FU and totally reverses 5-FU resistance in two 5-FU-resistant colorectal cancer cell lines *in vitro* (Wang *et al*, 2002). Thus, targeting NF-*κ*B may be a new measure to overcome TS inhibitor resistance.

High transcription factor activity in cancer cells is frequently induced by gene amplification and/or overexpression (Adams and Kaelin, 1996). In this study, we demonstrated that overexpression of NF-*κ*B p50 and p65 protein and mRNA was detected in four out of five drug-resistant cell lines. The overexpressed NF-*κ*B protein levels in these cell lines were in parallel with those of enhanced NF-*κ*B nuclear binding activity. Thus, the constitutive gene overexpression may be the main cause of the high NF-*κ*B nuclear activity in these cell lines. Although NF-*κ*B DNA-binding activity in drug-sensitive cancer cell lines could be induced by transient exposure to 5-FU, no alteration of NF-*κ*B protein levels was observed in these cell lines (Figure 3A, B). These results indicate that transient TS inhibitor exposure can only temporarily induce NF-κB activity at a post-translational level. The constitutive NF-*κ*B nuclear activity supported by gene overexpression is the intrinsic character of most TS inhibitor-resistant cancer cells. NF-*κ*B deregulation in drug-resistant cell lines is mainly at the transcriptional, translational, nuclear transporting and DNA-binding levels because no gene amplification was detected by Southern blotting. We have tested mRNA expression patterns in the same panel of cell lines using Atlas nylon membrane cDNA array system before and could not identify the mRNA overexpression of NF-*κ*B p65 that was spotted on the array membrane (Wang *et al*, 2001). This discrepancy may be caused by the sensitivity of the array system, especially for detecting the expression of low copy number genes.

Compared to other cell lines, the NF-*κ*B DNA-binding activity and protein expression patterns in H630_TDX_ cells are intriguing. NF-*κ*B nuclear DNA-binding activity in H630_TDX_ cells was highly upregulated. The NF-*κ*B proteins p50 and p65 in this cell line were similar to those in the parent cells. Apart from gene overexpression, high NF-*κ*B nuclear activity can be caused by enhanced nuclear translocation and DNA-binding affinity (Rayet and Gelinas, 1999). It has recently been reported that NF-*κ*B protein phosphorylation also plays an important role in the regulation of NF-*κ*B DNA-binding and transcriptional activity (Ghosh and Karin, 2002). Our results indicate that high constitutive NF-*κ*B activity in TS inhibitor-resistant cell lines may be caused by different molecular mechanisms.

I*κ*B degradation is thought to be a main reason for induction and maintenance of high nuclear NF-*κ*B activity (Chiao *et al*, 1994; Karin, 1999). I*κ*B*α* is the predominant member of the I*κ*B family. I*κ*B*α* degradation was involved in 5-FU-induced NF-*κ*B activation in 5-FU-sensitive cell lines (Wang *et al*, 2002). However, in this study, high NF-*κ*B activity in TS inhibitor-resistant cells was not conferred by degradation of I*κ*B*α*. High levels of I*κ*B*α* were detected in all drug-resistant cancer cell lines that demonstrated high nuclear NF-*κ*B activity (Figure 1D). It was reported that constitutive nuclear NF-*κ*B activity in two chemoresistant lymphoma cell lines was caused by aberrant activation of IKK's and overexpression of defective I*κ*B*α* (Krappmann *et al*, 1999). The role of I*κ*B*α* in NF-*κ*B signalling pathways in TS inhibitor-resistant cell lines remains to be elucidated.

## References

[bib1] Adams PD, Kaelin WG (1996) The cellular effects of E2F overexpression Curr Top Microbiol Immunol 208: 79–93 8575214 10.1007/978-3-642-79910-5_4

[bib2] Baldwin Jr AS (1996) The NF-kappaB and IkappaB proteins: new discoveries and insights. Annu Rev Immunol 14: 649–683 8717528 10.1146/annurev.immunol.14.1.649

[bib3] Baldwin Jr AS (2001) The transcription factor NF-kappaB and human disease. J Clin Invest 107: 3–6 11134170 10.1172/JCI11891 PMC198555

[bib4] Barkett M, Gilmore TD (1999) Control of apoptosis by Rel/NF-kappaB transcription factors. Oncogene 18: 6910–6924 10602466 10.1038/sj.onc.1203238

[bib5] Boonsong A, Marsh S, Rooney PH, Stevenson DA, Cassidy J, McLeod HL (2000) Characterization of the topoisomerase I locus in human colorectal cancer. Cancer Genet Cytogenet 121: 56–60 10958942 10.1016/s0165-4608(00)00242-9

[bib6] Burris H, Von Hoff D, Bowen K, Heaven R, Rinaldi D, Eckardt J, Fields S, Campbell L, Robert F, Patton S, Kennealey G (1994) A phase II trial of ZD1694, a novel thymidylate synthase inhibitor, in patients with advanced non-small cell lung cancer. Ann Oncol 5(Suppl 5): 133

[bib7] Chiao PJ, Miyamoto S, Verma IM (1994) Autoregulation of I kappa B alpha activity. Proc Natl Acad Sci USA 91: 28–32 8278379 10.1073/pnas.91.1.28 PMC42879

[bib8] Cunningham D, Zalcberg J, Smith IE et al (1994) Tomudex: a novel thymidylate synthase (TS) inhibitor with clinical antitumour activity in a range of solid tumours. Ann Oncol 5(Suppl. 8): 179 8777175 10.1093/oxfordjournals.annonc.a010546

[bib9] Cusack JCJ, Liu R, Baldwin Jr AS (2000) Inducible chemoresistance to 7-ethyl-10-[4-(1-piperidino)-1-piperidino]-carbonyloxycamptothecin (CPT-11) in colorectal cancer cells and a xenograft model is overcome by inhibition of nuclear factor-kappaB activation. Cancer Res 60: 2323–2330 10811101

[bib10] Cusack JCJ, Liu R, Houston M, Abendroth K, Elliott PJ, Adams J, Baldwin Jr AS (2001) Enhanced chemosensitivity to CPT-11 with proteasome inhibitor PS-341: implications for systemic nuclear factor-[kappa]B inhibition. Cancer Res 61: 3535–3540 11325813

[bib11] Ghosh S, Karin M (2002) Missing pieces in the NF-kappaB puzzle. Cell 109: S81–S96 11983155 10.1016/s0092-8674(02)00703-1

[bib12] Hohmann HP, Remy R, Scheidereit C, van Loon APGM (1991) Maintenance of NF-*κ*B activity is dependent on protein synthesis and the continuous presence of external stimuli. Mol Cell Biol 11: 259–266 1986224 10.1128/mcb.11.1.259-266.1991 PMC359616

[bib13] Jackman AL, Calvert AH (1995) Folate-based thymidylate synthase inhibitors as anticancer drugs. Ann Oncol 6: 871–881 8624289 10.1093/oxfordjournals.annonc.a059353

[bib14] Jackman AL, Kelland LR, Kimbell R, Brown M, Gibson W, Aherne GW, Hardcastle A, Boyle FT (1995) Mechanisms of acquired resistance to the quinazoline thymidylate synthase inhibitor ZD1694 (Tomudex) in one mouse and three human cell lines. Br J Cancer 71: 914–924 7537518 10.1038/bjc.1995.178 PMC2033796

[bib15] Karin M (1999) How NF-kappaB is activated: the role of the IkappaB kinase (IKK) complex. Oncogene 18: 6867–6874 10602462 10.1038/sj.onc.1203219

[bib16] Krappmann D, Emmerich F, Kordes U, Scharschmidt E, Dorken B, Scheidereit C (1999) Molecular mechanisms of constitutive NF-kappaB/Rel activation in Hodgkin/Reed–Sternberg cells. Oncogene 18: 943–953 10023670 10.1038/sj.onc.1202351

[bib17] Pahl HL (1999) Activators and target genes of Rel/NF-kappaB transcription factors. Oncogene 18: 6853–6866 10602461 10.1038/sj.onc.1203239

[bib18] Perkins ND (2000) The Rel/NF-kappa B family: friend and foe. Trends Biochem Sci 25: 434–440 10973057 10.1016/s0968-0004(00)01617-0

[bib19] Rayet B, Gelinas C (1999) Aberrant rel/nfkb genes and activity in human cancer. Oncogene 18: 6938–6947 10602468 10.1038/sj.onc.1203221

[bib20] Rustum YM, Harstrick A, Cao S, Vanhoefer U, Yin M-B, Wilde H, Seeber S (1997) Thymidylate synthase inhibitors in cancer therapy: direct and indirect inhibitors J Clin Oncol 15: 389–400 8996166 10.1200/JCO.1997.15.1.389

[bib21] Smith IE, Spielmann M, Bonneterre J, Namer M, Green M, Wandar HE Toussaint C, Azab M (1994) Tomudex (ZD1694), a new thymidylate synthase inhibitor with antitumour activity in breast cancer. Ann Oncol 5(Suppl. 5): 132

[bib22] Wang C-Y, Cusack JCJ, Liu R, Baldwin Jr AS (1999a) Control of inducible chemoresistance: enhanced anti-tumor therapy through increased apoptosis by inhibition of NF-kappaB. Nat Med 5: 412–417 10202930 10.1038/7410

[bib23] Wang C-Y, Guttridge DC, Mayo MW, Baldwin Jr AS (1999b) NF-kappab induces expression of the Bcl-2 homologue A1/Bfl-1 to preferentially suppress chemotherapy-induced apoptosis. Mol Cell Bio 19: 5923–5929 10454539 10.1128/mcb.19.9.5923 PMC84448

[bib24] Wang C-Y, Mayo MW, Baldwin Jr AS (1996) TNF- and cancer therapy-induced apoptosis: potentiation by inhibition of NF-kappaB. Science 274: 784–787 8864119 10.1126/science.274.5288.784

[bib25] Wang W, Marsh S, Cassidy J, McLeod HL (2001) Pharmacogenomic dissection of resistance to thymidylate synthase inhibitors. Cancer Res 61: 5505–5510 11454699

[bib26] Wang C-Y, Mayo MW, Korneluk RG, Goeddel DV, Baldwin Jr AS (1998) NF- [similar] B antiapoptosis: induction of TRAF1 and TRAF2 and c-IAP1 and c- IAP2 to suppress caspase-8 activation. Science 281: 1680–1683 9733516 10.1126/science.281.5383.1680

[bib27] Wang W, McLeod HL, Cassidy J (2002) Disulfiram inhibits 5-FU-induced NF-*κ*B activity and enhances cytotoxicity of 5-FU to human colorectal cancer cell lines. Bri J Cancer 86(Suppl 1): S88

[bib28] Wu MX, Ao Z, Prasad KVS, Wu R, Schlossman SF (1998) IEX-1L, an apoptosis inhibitor involved in NF-kappaB-mediated cell survival. Science 281: 998–1001 9703517 10.1126/science.281.5379.998

[bib29] Yamamoto Y, Gaynor RB (2001) Therapeutic potential of inhibition of the NF-kappaB pathway in the treatment of inflammation and cancer. J Clin Invest 107: 135–142 11160126 10.1172/JCI11914 PMC199180

